# Wrist-worn wearables based on force myography: on the significance of user anthropometry

**DOI:** 10.1186/s12938-020-00789-w

**Published:** 2020-06-12

**Authors:** Mona Lisa Delva, Kim Lajoie, Mahta Khoshnam, Carlo Menon

**Affiliations:** Menrva Research Group, Schools of Mechatronic Systems and Engineering Science, Simon Fraser University, Metro Vancouver, Unit 250, 13450 102nd Avenue, Surrey, BC V5A 1S6 Canada

**Keywords:** Wearable technology, Force myography, User anthropometry, Biosignal quality, Machine learning

## Abstract

**Background:**

Force myography (FMG) is a non-invasive technology used to track functional movements and hand gestures by sensing volumetric changes in the limbs caused by muscle contraction. Force transmission through tissue implies that differences in tissue mechanics and/or architecture might impact FMG signal acquisition and the accuracy of gesture classifier models. The aim of this study is to identify if and how user anthropometry affects the quality of FMG signal acquisition and the performance of machine learning models trained to classify different hand and wrist gestures based on that data.

**Methods:**

Wrist and forearm anthropometric measures were collected from a total of 21 volunteers aged between 22 and 82 years old. Participants performed a set of tasks while wearing a custom-designed FMG band. Primary outcome measure was the Spearman’s correlation coefficient (*R*) between the anthropometric measures and FMG signal quality/ML model performance.

**Results:**

Results demonstrated moderate (0.3 ≤|*R*| < 0.67) and strong (0.67 ≤ |*R*|) relationships for ratio of skinfold thickness to forearm circumference, grip strength and ratio of wrist to forearm circumference. These anthropometric features contributed to 23–30% of the variability in FMG signal acquisition and as much as 50% of the variability in classification accuracy for single gestures.

**Conclusions:**

Increased grip strength, larger forearm girth, and smaller skinfold-to-forearm circumference ratio improve signal quality and gesture classification accuracy.

## Background

Wearable technologies are now widely used to track and record movement related parameters [1]. For instance, electromyography (EMG) records muscle activity and force production occurring during movement, and can be used to provide the necessary commands for controlling external devices like computers [2, 3]. It is often used in rehabilitation protocols in research and in the clinic [4, 5]. However, EMG requires proper skin preparation and electrode placement, making it difficult for the general public to use in the home [6]. Inertial measurement units (IMUs) are small and able to detect limb acceleration, rotation and position [7], but are prone to signal noise and drift, as well as magnetic interference from home appliances and other devices [1]. Force myography (FMG), also referred to as topographic force mapping [8], residual kinetic imaging [9], and surface muscle pressure [10], records the volumetric changes that occur around a limb during muscle contraction. FMG has been used in multiple applications, including exoskeleton control [11–15], gait analysis [16], gesture identification [17–21], and rehabilitation [22, 23]. This technique has also been applied to assistive technology for amputees [11, 13] [24, 25] and stroke survivors [23]. Specifically, FMG does not require extensive skin preparation and specific electrode placement [8], nor does it require expertise for optimal implementation. Other advantages include increased signal stability over time for static gestures [12], robustness to external electrical interference and sweating [26], simpler signal processing than EMG datasets [17], and cost-effectiveness. These considerations are important for the deployment of FMG-based wearable devices in the general community.

Machine learning (ML) algorithms are recognized as powerful classification and pattern recognition tools to extract features from biosignals [27]. For example, FMG signals have been analyzed using ML-based techniques to monitor activities of both upper- and lower extremities for applications including discrete finger movement and hand gesture predictions [17, 18, 20, 21, 28, 29], continuous finger movement predictions [30], and gait monitoring [16]. Linear discriminant analysis, support vector machine, and random forest algorithms are examples of such implemented methods.

Due to the nature of FMG signal acquisition, user anthropometry can lead to confounding effects. For instance, the pressure caused by muscle volumetric changes during contraction is transmitted to FMG sensors through overlying connective tissue, subcutaneous fat and skin. Intuitively speaking, the mechanical properties of each of these body components can affect signal quality. This is particularly significant, as numerous changes in body composition occur throughout the lifespan [31]. Specifically, declines in muscle mass and muscular strength [32] might affect how gestures are discriminated by FMG, as there is a positive correlation between muscle cross-sectional area and strength [33]. In addition, the amount of subcutaneous adipose tissue decreases with age [34], and connective tissue and skin become thinner and less elastic [35], all of which can influence how muscular forces are transmitted. Differences in body composition between biological males and females are also evident [36].

The aim of this preliminary study is to identify anthropometric measures that might systematically influence FMG signal acquisition and ML algorithm performance, and to quantify the extent of the variability introduced. This study is the first of its kind to consider the characteristics and effects unique to using FMG across as wide range of user characteristics.

## Methods

### Participants

Twenty-one participants (11 males and 10 females) were recruited from Simon Fraser University and the general community. Inclusion criteria were being able to follow instructions in English and perform the required gestures/tasks to completion. Exclusion criteria were self-identified neurological impairments and musculoskeletal barriers to functional movements. The study was approved by the Office of Research Ethics at Simon Fraser University, and all participants provided informed written consent. Testing took place at SFU and Confederation Seniors Centre in British Columbia, Canada.

### Anthropometric measures

To investigate and identify the participant-specific measurable features that might cause variability in FMG signals, the following anthropometric measures were considered. Values obtained are reported in Table 1.

**Table 1:** Participant demographics and anthropometric measures

Measure	Value
Age (years)	39.50 (21.09)
Weight (kg)	78.06 (14.64)
Height (m)	1.70 (0.13)
Body mass index (kg/m^2^)	26.81 (4.25)
Wrist circumference (cm)	17.19 (1.68)
Forearm circumference (cm)	26.09 (3.24)
Forearm length (cm)	26.74 (1.99)
Skinfold thickness (cm)	0.93 (0.39)
Ratio: skinfold thickness to forearm circumference (unitless)	0.03 (0.02)
Ratio: wrist circumference to forearm circumference (unitless)	0.67 (0.07)
Maximum grip strength (kg)	21.32 (9.69)

Values are presented as *µ* ($\sigma$^2^), where *µ* is the mean and $\sigma$^2^ is the standard deviation

#### Limb length and circumference

Standard positioning for limb length and circumference measurements was a neutral shoulder, 90° flexed elbow, neutral forearm, neutral wrist orientation, and relaxed hand. Forearm length was measured from the olecranon process to the ulnar styloid process and its circumference was measured at the widest part of the forearm. The length of the upper arm was measured from the acromial process to the olecranon process. Wrist circumference was measured within 1 inch proximal to the head of the radius and ulna, i.e., the thinnest part of the wrist. All measurements were done using a standard tape measure and were rounded to the nearest millimeter.

#### Skinfold thickness

Skinfold thickness was measured from the anterior aspect of the forearm, approximately at its widest part. Methods for taking skinfolds were taken from [42]. In brief, the skin was firmly grasped between the first three digits. The jaws of the calipers were then placed approximately 1 cm from where the skin was grasped, and the skin was released for measurement. An analog Slim Guide Skinfold Caliper (Creative Health Products, Ann Arbor, MI) was used for this purpose, and all measurements were rounded to the nearest millimeter.

#### Active range of motion

Active range of motion (ROM) of the wrist and the forearm were captured using a goniometer (Jamar Plus + Digital 8″ Goniometer, Patterson Medical, Cedarburg, WI). Wrist ROM for flexion and extension was measured while the shoulder was in neutral position, with the elbow flexed at 90° and the forearm held in neutral orientation. Participants were instructed to actively flex or extend their wrist to its fullest capacity, and measurements for each motion were taken as the smallest relative angle between the second meta-carpal and the central axis of the radius. Measurements were rounded to the nearest degree.

Forearm ROM for pronation and supination was measured while the shoulder was in neutral position, with the elbow flexed at 90° and the wrist in neutral orientation. Participants were instructed to hold a pencil in a closed fist while actively pronating or supinating the forearm to its fullest capacity. Measurements for each motion were taken as the smallest relative angle between the starting and end positions of the length of the pencil. Measurements were rounded to the nearest degree.

#### Grip strength

Grip strength tests were performed with the shoulder and wrist in neutral positions and the elbow flexed at 90°. A non-deformable digital hand dynamometer (HD-BTA, Vernier Software & Technology, Beaverton, OR) was used to measure grip strength. Participants were instructed to the hold the hand dynamometer in a closed fist and to squeeze it with maximum effort (maximum voluntary contraction) for approximately 3 s. This test was repeated 3 times, and the average value was rounded to the nearest 0.01 kg [43].

All measurements were performed on the right side before the start of the protocol.

### Study protocol

Participants were asked to use their right hand to perform instructed movements while seated on a chair of standard height and depth. This section describes the selected movements, the experimental task, and the procedure for data collection and analysis in detail.

### Selected movements

**Fig. 1:**
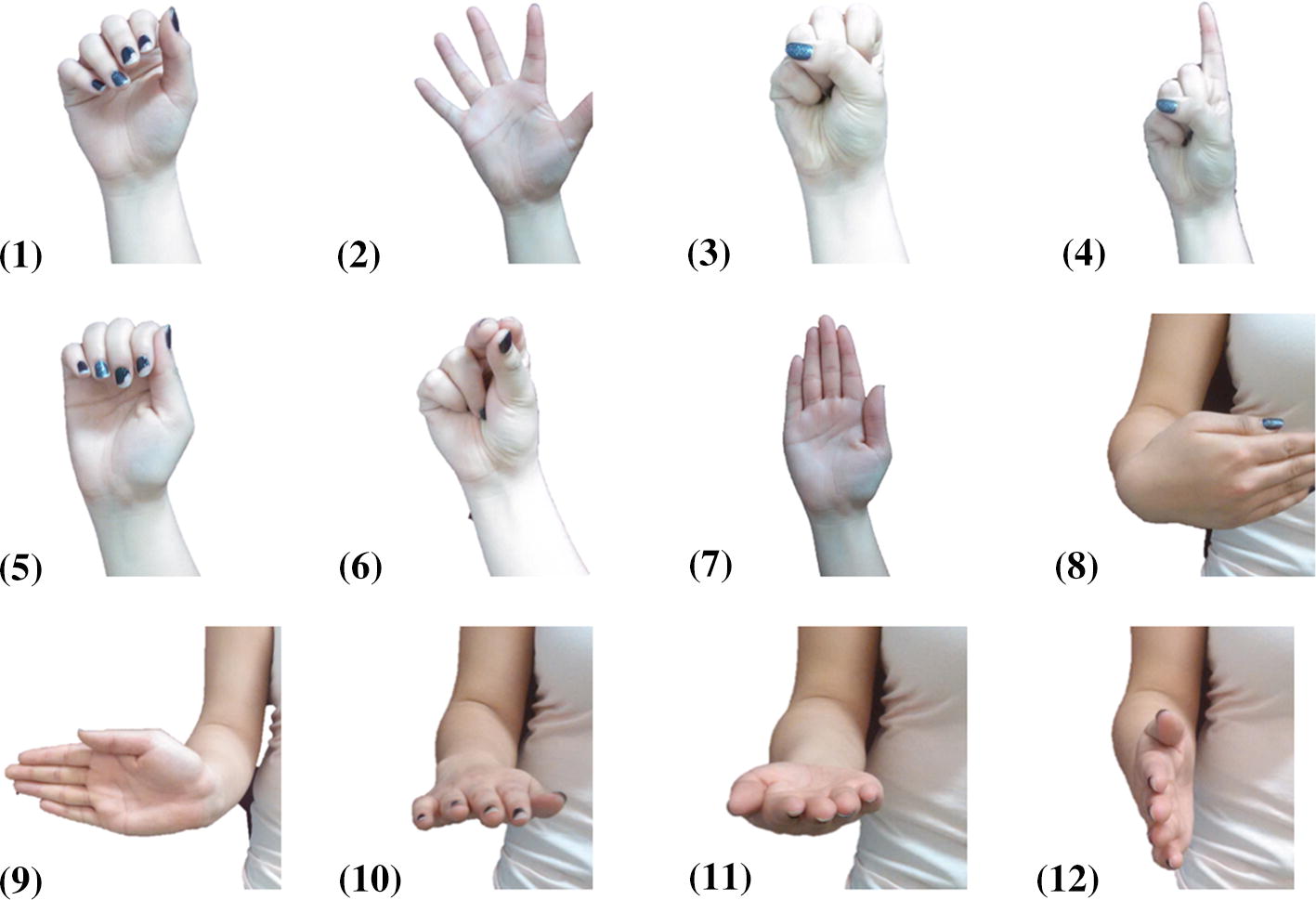
Hand gestures and wrist orientations: (1) relax; (2) open; (3) close; (4) point; (5) key (lateral pinch); (6) tripod pinch; (7) straight; (8) wrist flexion; (9) wrist extension; (10) forearm pronation; 11) forearm supination; (12) neutral wrist/forearm

Common hand gestures and wrist/forearm orientations for performing activities of daily living [44] were selected as the movement set performed by each participant. The selected hand gestures (Fig. 1 1–7) included:

1. Relax: fingers and thumb are not actively engaged in flexion or extension,
2. Open: fingers and thumb are fully extended and fully abducted,
3. Close: a fist with the buttressing of the distal tips of the phalanges against the central palm and buttressing of the thenar eminence and thumb against the dorsal surfaces of digits 2 and 3 [45] or the lateral aspect of the 2nd digit,
4. Point: only the 2nd digit (index finger) is fully extended, and the pad of the thumb is resting on the lateral aspect of the 3rd digit,
5. Key: the pad of the thumb is in contact with the proximal interphalangeal joint of the 2nd digit,
6. Tripod: the pads of the thumb, 2nd, and 3rd digits are in contact.
7. Straight: hand is open, and fingers and thumb are extended and adducted.

For each of the wrist and forearm orientations (flexion, extension, pronation, supination, and neutral), unless otherwise indicated, participants were instructed to keep the fingers and thumb fully extended and adducted **(**Fig. 1 8–12). It is also worthwhile noting that the straight hand gesture (Fig. 1 7) and the neutral wrist/forearm orientations (Fig. 1 12) are similar. Therefore, when considering a combined set of hand gestures and wrist/forearm orientations, 11 distinct gestures were included.

**Fig. 2:**
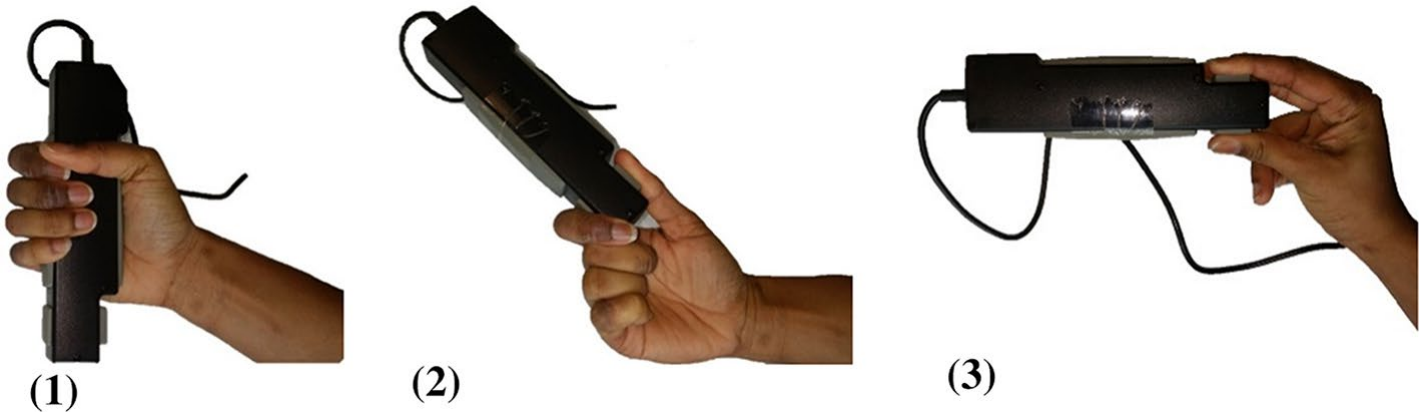
Grip strength measurements: (1) cylindrical grip, (2) key grip, (3) tripod grip

To evaluate grip strength, participants were instructed to grip a hand dynamometer with the following grips: cylindrical, key and tripod (Fig. 2).

### Experimental task

Three groups of tasks were defined based on the selected hand gestures and wrist/forearm orientations (Fig. 1):

1. Static singleton gestures: participants were instructed to perform each of the hand gestures and wrist/forearm orientations individually, for a total of 11 different classes (gesture groups). Participants performed 5 randomized repetitions of each static gesture for approximately 7 s each.
2. Static compound gestures: participants were instructed to simultaneously perform a hand gesture and a wrist/forearm orientation. For example, for ‘point_supination’, participants were instructed to simultaneously point the hand (Fig. 1 4) and supinate the forearm (Fig. 1 11). Participants performed 5 randomized repetitions of each possible combination for approximately 7 s each.
3. Dynamic motions: participants were instructed to either move between two ROM extremes (i.e., from wrist flexion to extension), or to grip while applying minimal to maximal effort. Participants performed one repetition of each dynamic motion for 60 s. Table 2 provides an overview of the specific gestures that constitute each group of tasks.

**Table 2:** Tasks completed by participants in experimental protocol

Dynamic motions	Static singleton gestures	Static compound gestures	
Flexion < - > extension Pronation < - > supination Cylindrical grip, squeeze and relax Tripod grip, squeeze and relax Key grip, squeeze and relax	Relax Open Close Point Key Tripod Straight Flexion Extension Pronation Supination Neutral	All possible combinations of hand gestures and wrist/forearm orientations	
		Hands gestures Relax Open Close Point Key Tripod	Wrist orientations Flexion Extension Pronation Supination Neutral

Participants were in a relaxed posture before the performance of each gesture/motion.

### Data collection

#### Force myography (FMG) band

A custom force myography (FMG) band was designed to quantify limb volumetric changes while performing the selected movements. Force-sensitive resistors (FSRs) were selected for FMG acquisition due to their low-profile dimensions, flexibility, cost-effectiveness, wide-spread availability, and their ease of implementation into a portable and wireless device. The band used in this study utilized 16 FSRs (FSR 400, Interlink Electronics, Inc., Los Angeles, CA) in a staggered design. An FSR consists of a polymer thick film (PTF) circuitry printed on a flexible substrate with a variable force–resistance relationship. FSRs were implemented in series with a 4.6-kΩ resistor, and supplied with a voltage of 3.7 V. An ATMega328 microprocessor (Microchip Technology, Chandler, AZ) was used to facilitate data collection and transmission. Each FSR was sampled at approximately 10 Hz, with raw analog values converted to a digital signal ranging from 0 to 1023 (0.00361 V/bit). Digital values were time stamped and transmitted to an on-site computer via serial connection, then saved onto a .txt file for offline processing.

**Fig. 3:**
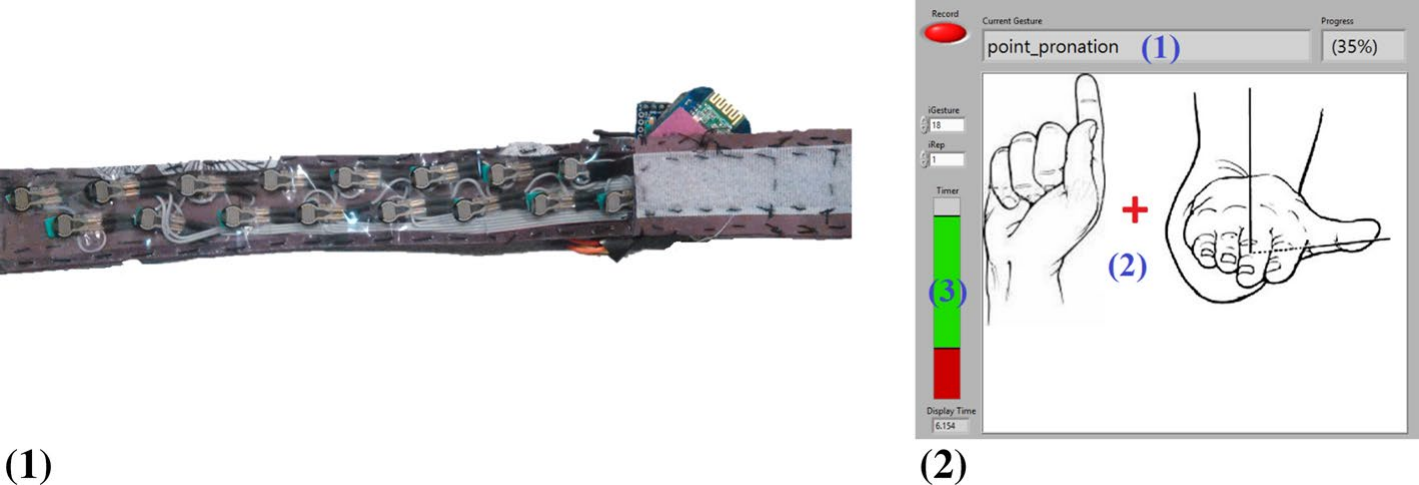
(1) Staggered placement of the FSRs on a flexible non-elastic backing. (2) Instruction interface: (1) current instructed gesture, (2) instruction image, (3) gesture timer

The FSRs were backed with Flex foam and secured to the interior of the band, which was lined with cellulose acetate, a flexible and non-elastic material commonly used in overhead projector film transparencies. This material was used to facilitate a firmer contact between the FSR and the skin, while allowing the band to conform to the shape of the wrist. Participants donned the FMG band on the wrist, 1 to 1.5 in. proximal to the radial and ulnar styloid process surface landmarks. The placement of FSRs on the band is shown in Fig. 3 1. The placement of the band on the right wrist was similar to that shown in [46].

Instructions regarding the gestures were presented as images via a visual interface (Fig. 3 2) designed in LabVIEW 2014 and displayed in real time on a monitor positioned between eye level and desk level. When necessary, the investigator demonstrated the hand gesture desired at the time and/or corrected the participants’ hand gesture to ensure uniformity between all participants.

#### Data processing

FMG signals were collected while participants performed instructed hand and wrist gestures (Table 2), then normalized based on the global minimum and maximum of acquired data. We then generated several ML models to establish trends of FMG behavior that were persistent despite selected models and chosen parameters. More specifically, four multi-output ML models, namely artificial neural network (ANN), extreme machine learning (ELM), linear discriminant analysis (LDA), and support vector machine (SVM), were generated and trained using built-in machine learning functions in MATLAB R2016a.

### Gesture classification using machine learning

All ML models were trained using data collected while performing static singleton gestures (Table 2). These models previously demonstrated good accuracy in hand gesture classification based on FMG data [17, 47, 48]. ML models were then used to identify the singleton gestures performed during compound gestures or dynamic motions. For instance, during the ‘Wrist Flexion/Extension’ task, the model output is expected to be either flexion, neutral, or extension, based on the orientation of the hand with respect to the forearm. An exploratory analysis considering 11 static gestures **(**Table 2) as the ML model output showed that training the ELM model using raw FMG data results in higher classification accuracy, while the classification accuracy of SVM, LDA, and ANN models is higher if normalized FMG data are used as the model input.

#### Outcome measures

The primary outcome measure for this study was the correlation between anthropometric measures (“Grip strength” section) and FMG signal quality/ML model performance. Spearman’s correlation coefficient (*R*) was used to evaluate such a correlation: a smaller *R* value, where |*R*| < 0.33, was considered a weak correlation, a medium range *R* value, 0.33 ≤ |*R*| < 0.67, represented a moderate correlation, and 0.67 ≤ |R| demonstrated a strong correlation.

Anthropometric measures considered were grip strength, skinfold thickness, and wrist and forearm circumferences. Moreover, several measurements were combined to create the following measures:

1. Ratio of skinfold thickness to forearm circumference (skinfold:forearm), which is an indicator of the ratio of bone/muscle to skin/adipose tissue and has a value in the range of [0,1],
2. Ratio of wrist circumference to forearm circumference (wrist:forearm) which is an indicator of muscle bulk around the forearm and has a value in the range of [0,1].

The quality of acquired FMG signals was quantified using two measures:

1. separability of gesture class clusters: to quantify the linear separateness of the data clusters used in classification, the separability index, $J_{B/W}$, was defined as
  1 $$ J_{B/W} = {\text{trace}}\left( {S_{W}^{ - 1} S_{B} } \right), $$

where

2 $$ S_{W} = \mathop \sum \limits_{i = 1}^{{n{\text{Classes}}}} P_{i} S_{i} \;{\text{and}}\;S_{B} = \mathop \sum \limits_{i = 1}^{{n{\text{Classes}}}} P_{i} \left( {m_{i} - m} \right)\left( {m_{i} - m} \right)^{T} $$

are within-class and between-class scatter matrices, respectively, with

I. $$ \omega_{i} $$: class label
II. $m_{i}$: mean of class $\omega_{i}$
III. $K_{i}$: number of samples in class $\omega_{i}$
IV. $m$: overall mean
V. $K$: overall number of samples
VI. $$ P_{i} $$: the a priori probability of class $$ \omega_{i} = {\raise0.7ex\hbox{${K_{i} }$} \!\mathord{\left/ {\vphantom {{K_{i} } K}}\right.\kern-0pt} \!\lower0.7ex\hbox{$K$}} $$
VII. $S_{i}$: scatter (covariance) matrix for class $\omega_{i}$

Intuitively, the optimal separability, i.e., larger values of $J_{B/W}$, is achieved by maximizing the between-class variance and minimizing the within-class variance.

2. Sensitivity: this measure was defined as the magnitude of FMG response to incremental changes in orientation. Sensitivity was represented as the root mean square (RMS) of change in the FMG signal magnitude from baseline values across all sensors.

The performance of the developed machine learning models in detecting static and dynamic gestures was quantified using the following measures:

1. In the case of static gestures, the model performance was represented by accuracy, i.e., ratio of correctly classified gestures over total number of performed gestsures.
2. In the case of dynamic motions, the model performance was represented by the following measures: (1) the number of correct classes, (2) whether these classes overlapped with the training region. For instance, during the ‘Wrist Flexion/Extension’ task, the number of correct classes is three (flexion, neutral, and extension), and as long as the wrist is flexed, the output class is expected to be ‘flexion’.

## Results

### Participants’ anthropometric measures and range of motion

Anthropometric measures were determined for each participant as detailed in “Methods” section. Table 1 summarizes the average and standard deviation for those measurements.

During a number of dynamic motions, e.g., wrist flexion/extension, participants were asked to move through their full range of motion. The ROM values for different joints, measured at the beginning of the data collection session, are listed in Table 3.

**Table 3:** Range of motion (ROM) across all dynamic tasks

	Female	Male
Non-senior (19–59 years old)		
Wrist flexion	96.00° (12.68°)	72.49° (11.16°)
Wrist extension	− 55.42° (5.90°)	− 55.40° (11.95°)
Forearm pronation	59.68° (22.74°)	70.00° (13.80°)
Forearm supination	− 64.62° (5.66°)	− 67.84° (13.37°)
Wrist full range	151.42° (10.80°)	127.90° (16.62°)
Forearm full range	124.30° (20.02°)	137.84° (18.65°)
Senior (60 + years old)		
Wrist flexion	86.79° (10.36°)	89.21° (13.05°)
Wrist extension	− 54.93° (4.59°)	− 51.12° (9.32°)
Forearm pronation	82.21° (14.18°)	72.51° (10.69°)
Forearm supination	− 60.20° (14.15°)	− 56.83° (23.84°)
Wrist full range	141.72° (14.66°)	140.33° (3.73°)
Forearm full range	142.41° (16.14°)	129.34° (13.15°)

Positive (+) values for wrist flexion and forearm pronation, while using (−) values for wrist extension and forearm supination. Values are presented as *µ* (*σ*^2^), where *µ* is the mean and *σ*^2^ is the standard deviation

### Impact of anthropometric measures on FMG signal quality

#### Correlation between anthropometric measures and separability

At neutral wrist, the mean (standard deviation) separability of FMG clusters across the seven hand gestures **(**Fig. 1 1–7) was 124.79 (96.35).

With a straight hand gesture, the mean (standard deviation) separability of FMG clusters across the five wrist/forearm positions **(**Fig. 1 8–12**)** was 76.21 (41.28). Mean correlations between user anthropometry and data cluster separability are tabulated in Table 4.

**Table 4:** Correlation between anthropometric measures and separability of gesture classes

Variable	Correlation coefficient (R)	
	Separability of hand classes	Separability of wrist classes
Grip strength	0.53	0.34
Skinfold thickness	− 0.20	− 0.16
Skinfold:forearm	0.57	0.40
Wrist:forearm	− 0.45	− 0.42
Wrist circumference	− 0.50	− 0.43
Forearm circumference	− 0.40	− 0.13

#### Correlation between anthropometric measures and sensitivity

Mean correlations between user anthropometry and sensitivity of acquired FMG signals are tabulated in Table 5. Grip strength and ratio of skinfold thickness to forearm circumference demonstrated the greatest relationship with FMG responsiveness.

**Table 5:** Correlation between anthropometry and sensitivity

Variable	Correlation coefficient (*R*)
Grip strength	0.55
Skinfold thickness	− 0.48
Skinfold:forearm	− 0.55
Wrist:forearm	− 0.39
Wrist circumference	0.13
Forearm circumference	0.33

### Impact of anthropometric measures on the performance of ML models

#### Correlation between anthropometric measures and model accuracy in classification of static gestures

To train the selected ML models, data collected from each participant were randomly split such that 60% of the collected data corresponding to each gesture were assigned to the training set, and the remaining 40% was used for testing the trained models. Mean training accuracies were 88.06, 90.38, 99.08, and 99.92 for SVM, LDA, ELM, and ANN, respectively. These models achieved mean testing classification accuracies of 87.95, 89.96, 98.89, and 99.82, respectively. It should be noted that an accuracy of 100% demonstrates perfect classification performance. Correlations between anthropometric measures and testing accuracy of considered ML models are tabulated in Table 6. Results show that the accuracy of ELM and NN models weakly correlated with anthropometric measures. However, the skinfold thickness and ratio of skinfold thickness to forearm circumference highly affected the accuracy of SVM and LDA models, while other anthropometric measures had moderate-to-low correlations with the accuracy of these models.

**Table 6:** Correlation between anthropometric measures and gesture/orientation classification accuracy (*SVM* support vector machine, *LDA* linear discriminant analysis, *ELM* extreme learning machine, *NN* neural network)

Variable	SVM	LDA	ELM	NN
Grip strength	0.44	0.50	0.18	0.24
Skinfold thickness	− 0.66	− 0.71	− 0.22	− 0.20
Skinfold:forearm	− 0.68	− 0.71	− 0.28	− 0.24
Wrist:forearm	− 0.57	− 0.46	− 0.23	− 0.28
Wrist circumference	− 0.14	− 0.11	0.03	− 0.03
Forearm circumference	0.23	0.13	0.17	0.12

#### Correlation between anthropometric measures and model performance in classification of dynamic tasks

**Fig. 4:**
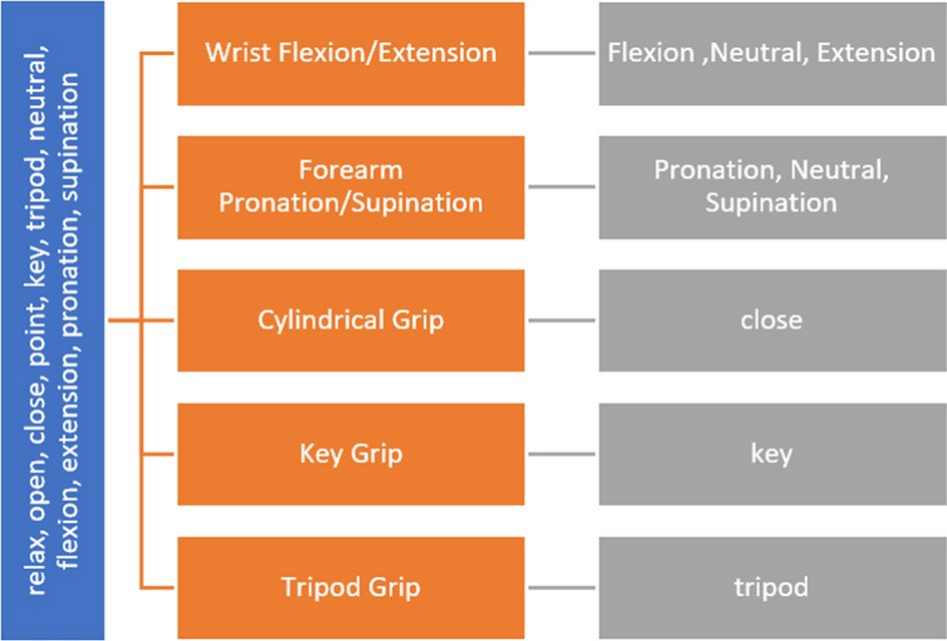
Schema for exploring gesture identification during non-static activities

The ML models trained for classification of static gestures were applied to the dynamic tasks presented in Table 2. Figure 4 shows the static gestures used to train models (blue box), non-static tasks onto which the trained models were applied (orange boxes), and the expected classification outputs based on the considered static gestures (gray boxes).

**Fig. 5:**
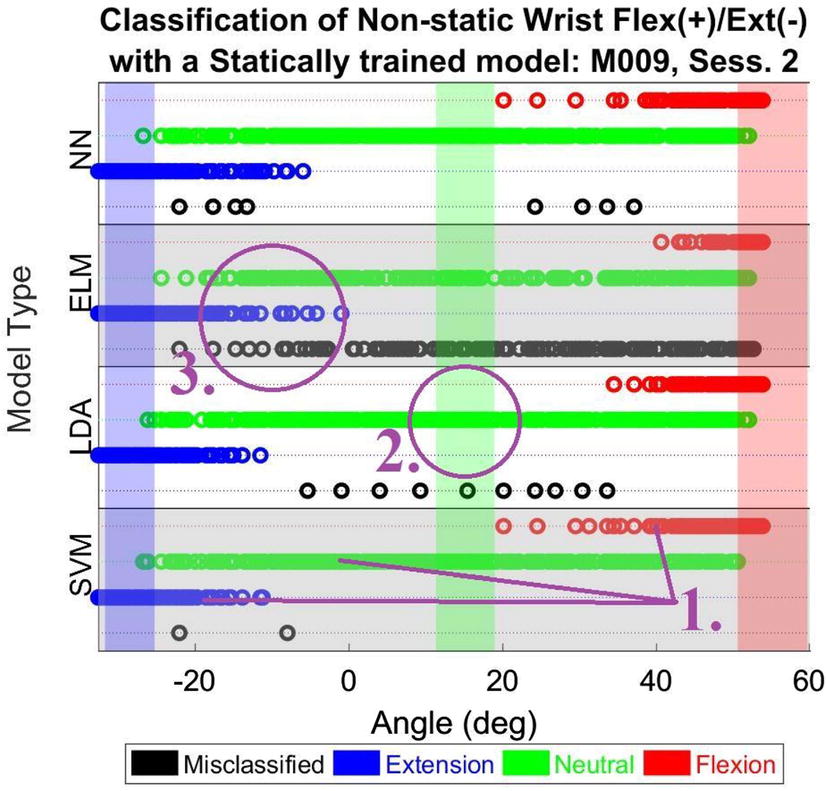
Performance of the models trained with static gestures in classifying dynamic wrist motion. Circular markers indicate the output of each model. Positive (+) values are assigned to wrist flexion and forearm pronation, while negative (−) values are assigned to wrist extension and forearm supination

Figure 5 shows an example of the performance of trained models in identifying the dynamic wrist extension/flexion motion. In the shown case, the number of classes is three: flexion, neutral, extension (marked by number 1 on Fig. 5). The identified classes overlap with corresponding actual wrist orientations in regions where circular markers have the same color as the background shade (an example is marked by a purple circle numbered 2 on Fig. 5). Moreover, the variability within each cluster is characterized by the relative number of model outputs that did not correspond to the actual wrist orientation (an example is marked by a purple circle numbered 3 on Fig. 5).

**Fig. 6:**
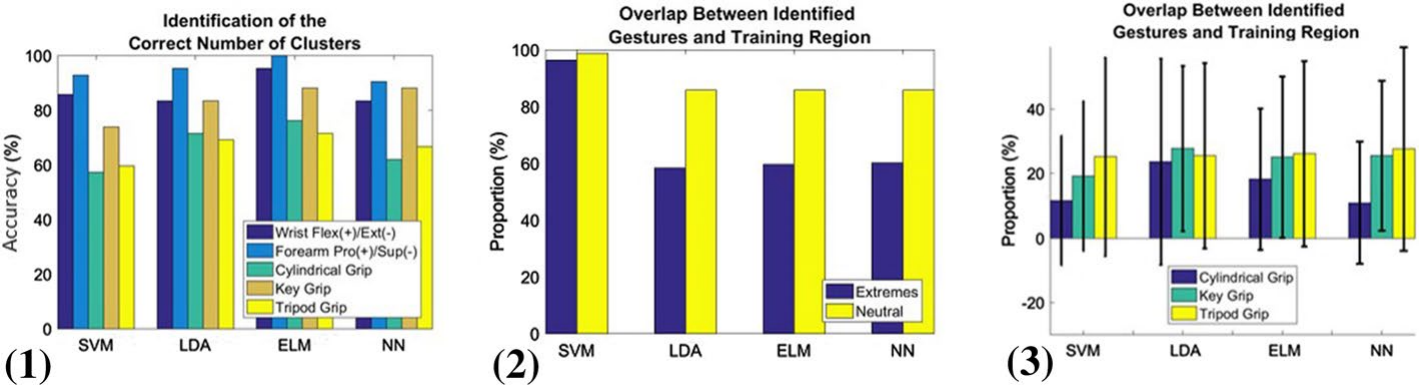
(1) Accuracy of trained models in identifying the correct number of static gestures occurring during the dynamic tasks. (2) Proportion of correct overlap between identified gestures and actual arm/wrist orientation in wrist flexion/extension and forearm pronation/supination motions. (3) Proportion of correct overlap between identified gestures and corresponding actual gesture in cylindrical grip, key grip, and tripod grip during dynamic tasks (*SVM* support vector machine, *LDA* linear discriminant analysis, *ELM* extreme learning machine, *NN* neural network)

Considering the data collected from all participants performing the dynamic tasks, Fig. 6 1 shows the accuracy of trained models in identifying the correct number of gestures for each task. Generally speaking, the correct number of gestures was correctly identified in at least 57% of cases across different tasks and trained models. Moreover, the number of gestures corresponding to dynamic wrist flexion/extension motion and forearm pronation/supination motion was correctly identified in more that 80% of cases using any of the trained models.

Figure 6 2 shows the proportion of correct overlap between the identified gestures and the corresponding actual orientation (training region) in wrist flexion/extension and forearm pronation/supination motions. Defining ‘Extremes’ as the fully flexed/extended state for the wrist and the fully pronated/supinated for the forearm, and ‘Neutral’ as the neutral wrist and neutral forearm position, the percentage of correct overlap is higher in the ‘Neutral’, reaching 96% using the trained SVM model. In the case of ‘Extremes’, SVM shows a high overlap percentage of 93%.

Figure 6 3 shows that continuous identification of close, key, and tripod grips during dynamic cylindrical grip, key grip, and tripod grip tasks is quite poor. This is indicated by low correct overlap (between 11% and 28%) between the identified gesture and its actual counterpart. Such a poor performance might be attributed to the participants’ handling of an object (a hand dynamometer) in the dynamic tasks, which was not present when performing the static gestures

All model performance measures reported in this section had low correlations (|*R*| < 0.3) with anthropometric measures.

### Additional results

Although this study was not focused on the effects of age and gender on the outcome measures, we investigated further. Specifically, participants were divided into two age groups, namely seniors (above 60 years old) and non-seniors (between 19 and 60 years old). Table 7 provides age- and gender-specific details of participant demographics and anthropometric measures. Student’s *t* test and ANOVA were implemented to reveal whether age and gender affect the quality of acquired FMG signals and the performance of the developed machine learning models. This analysis showed age-associated differences in the accuracy of classification, and in the separability of hand gestures and wrist gestures (*p*-value < 0.005). However, it should be noted that the number of participants was not balanced in age and gender groups (Table 7). Therefore, these results might have been skewed. This aspect is addressed further in the Discussion.

**Table 7:** Details of participant demographics and anthropometric measures based on age and gender

	Non-senior (19–59 years old)		Senior (60 + years old)	
	Female	Male	Female	Male
Number	6	9	4	2
Age (years)	26.25 (2.44)	27.11 (3.55)	74.75 (5.44)	64.50 (4.95)
Weight (kg)	65.40 (14.71)	87.11 (9.25)	74.50 (15.51)	82.41 (3.41)
Height (m)	1.61 (0.04)	1.83 (0.08)	1.59 (0.07)	1.65 (0.07)
Body mass index (kg/m^2^)	25.01 (4.74)	26.12 (3.17)	29.31 (5.57)	30.30 (1.34)
Wrist circumference (cm)	15.92 (1.88)	17.72 (0.97)	16.88 (1.80)	19.25 (0.35)
Forearm circumference (cm)	24.33 (2.82)	27.50 (3.82)	25.00 (1.78)	27.25 (0.35)
Forearm length (cm)	25.17 (1.57)	27.89 (1.54)	25.63 (1.80)	28.50 (0.71)
Skinfold THICKNESS (cm)	0.99 (0.23)	0.66 (0.25)	1.25 (0.51)	1.30 (0.42)
Ratio: skinfold thickness to forearm circumference (unitless)	0.04 (0.01)	0.02 (0.01)	0.05 (0.02)	0.05 (0.02)
Ratio: wrist circumference to forearm circumference (unitless)	0.65 (0.04)	0.66 (0.10)	0.68 (0.05)	0.71 (0.02)
Maximum grip strength (kg)	16.11 (4.43)	29.83 (7.55)	10.54 (2.72)	20.24 (4.45)

Values are presented as *µ σ*^2^), where *µ* is the mean and *σ*^2^ is the standard deviation

## Discussion

Force myography is a non-invasive method to measure volumetric changes in muscle cross-sectional area, which are transmitted through tissue. The resultant surface pressure changes can be used to track and identify movements and gestures. Due to the nature of force transduction through underlying tissue, user anthropometry may influence the quality of FMG signal acquisition and FMG-based modeling. Understanding how anthropometry can confound FMG performance is important for the creation of robust user-centered designs for wearable technologies. This exploratory study investigated the relationship between user anthropometric measures and FMG acquisition/modeling, and further quantified its magnitude.

With respect to FMG sensitivity, the greatest significant contributors to variability in FMG responsiveness were ratio of skinfold thickness to forearm circumference (*R* = − 0.55), and grip strength (*R* = 0.55). Grip strength is an anthropometric measure directly related to muscle fiber cross-sectional area. As FMG measures the volumetric changes that occur with activity, it is understandable that lower grip strengths would result in lower magnitudes of changes, which is supported by our results. When performing static gestures, increased grip strength was associated with improved hand gesture separability (*R* = 0.53). The relationship between grip strength and wrist motions was not as strong (*R* = 0.34). This difference is attributed to the muscle–tendon organization of the forearm and recruitment during wrist/hand movements. In the distal forearm, the anterior flexor compartment is composed of tendons responsible for finger flexion. Relative to motions of the wrist, these tendons are more engaged in the cylindrical grip during grip strength measurements, as well as in 5 of the 7 hand gestures, hence the stronger correlation. Despite the differences in the separability of wrist gestures versus hand gestures, the strong relationship between grip strength and gesture classification accuracy has implications for using FMG with certain populations.

Another variable related to muscle cross-sectional area and grip strength was the ratio of wrist to forearm circumference. Previous studies show a correlation between grip strength and forearm circumference [37, 38]. In the present study, the greater the forearm circumference relative to the wrist circumference, the better the improvements in sensitivity to change (*R* = − 0.39), which further highlights the significance of strength, as well as the underlying musculoskeletal bulk in FMG signal acquisition and gesture classification.

The correlation between the ratio of skin fold thickness to forearm circumference and FMG sensitivity had a moderate and negative correlation (*R* = − 0.55). As non-rigid structures, the skin and subcutaneous fat act as dampeners of the forces produced by muscle fibers as they are transmitted to the FSR sensors. Thus, an increase in adipose tissue likely increases the dampening effect on FMG signals and decrease the discriminability of gestures. This ratio also takes into consideration the combined effects of the skin/fat dampening and muscle cross-sectional area. A high ratio of skinfold thickness to forearm circumference also strongly correlated with lower classification accuracy, i.e., *R* = − 0.68 and − 0.71 for SVM and LDA models, respectively. Moderate relationships were observed with gesture separability for hand and wrist gestures, with *R* = 0.57 and *R* = 0.40, respectively.

Using models trained with static gestures to identify expected similar gestures performed during non-static activity presented moderate-to-high success (Fig. 6 1). However, this performance was not consistent throughout the full range of motion or effort and was not necessarily related to training conditions. Clusters of correctly identified activity were more consistent at the extremes of movements, but were least likely to overlap with the training region for that intended gesture. At neutral wrist/forearm, there was a high degree of variability and misclassification, however it is surmised that this is due to the inherently low separability of hand gestures. When the models were trained by performing hand gestures without objects, there was a low probability of correctly identifying that gesture in the presence of an object (< 27%). The performance of static models during non-static activity had little to no relationship with anthropometry measures and is therefore more likely related to the nature of the machine learning paradigm itself.

The identification of anthropometric measures that can influence FMG signal acquisition quality and modeling accuracy has the potential to enhance the development of FMG technology. It is recommended to explore these factors as weighting factors for classification and regression algorithms. In addition, this knowledge would be beneficial for sensor selection and calibration in future designs. However, the authors acknowledge that the current study has limitations. First, the number of participants is relatively low, and more participants would increase statistical power. In addition, the protocol was performed using the right arm, regardless of hand dominance. Grip strength has a documented relationship with hand dominance [39], which was not addressed in the current work. It is recommended that future iterations of this work be performed on both dominant and non-dominant hands and evaluate intra-subject variances.

The participants recruited for this study covered a wide age range (Table 7). The emphasis of this study was not on age and gender effects, although previous research reported a relationship between age, gender and weight, and anthropometry measures such as skinfold thickness [40]. Given the potential for FMG technology to be used as an assistive tool for senior populations [41], we thus encourage future studies to consider the effects of age and gender on the relationship between anthropometric measures and FMG signal acquisition quality and modeling accuracy.

## Conclusions

We presented data and analysis identifying and quantifying how individual differences in anthropometry measures can affect the quality of FMG signal acquisition and FMG-based modeling. Sixteen force-sensing resistors were arranged as a grid in a portable and wearable band to track hand gestures and wrist/forearm orientations. Participants perform a pre-selected set of gestures commonly used to perform activities of daily living. The protocol as presented identified three key anthropometric measures, namely grip strength, ratio of wrist to forearm circumference, and ratio of skinfold thickness to forearm circumference, as having moderate (0.3 < |*R*| < 0.6) influence on FMG signal acquisition and modeling. Increased grip strength, larger forearm girth, and smaller skinfold-to-forearm circumference ratio were shown to improve signal quality and hand gesture classification accuracy during static and dynamic conditions. These results have implications for the design of FMG technology for select populations like seniors, as well as guidelines for algorithm development. Future research could consider anthropometric characteristics as features in classification/regression algorithms and compare FMG to other myography modalities, while also considering the effect of age and gender.

## Data Availability

The datasets used and/or analyzed during the current study are available from the corresponding author on reasonable request.

## Abbreviations

EMG: Electromyography
IMU: Inertial measurement unit
FMG: Force myography
ML: Machine learning
ROM: Range of motion
FSR: Force-sensitive resistors
PTF: Polymer thick film
ANN: Artificial neural network
ELM: Extreme machine learning
LDA: Linear discriminant analysis
SVM: Support vector machine
RMS: Root mean square
